# Supplementary material for: SAMP-Score: a morphology-based machine learning classification method for screening pro-senescence compounds in p16 positive cancer cells
Source: Aging (Albany NY). 2025 Oct 30;17(11):2688–716. doi: 10.18632/aging.206333 (PMC12822720; doi:10.18632/aging.206333)
Supplement: Supplementary Figures [file aging-17-11-206333-s001.pdf]

SUPPLEMENTARY FIGURES

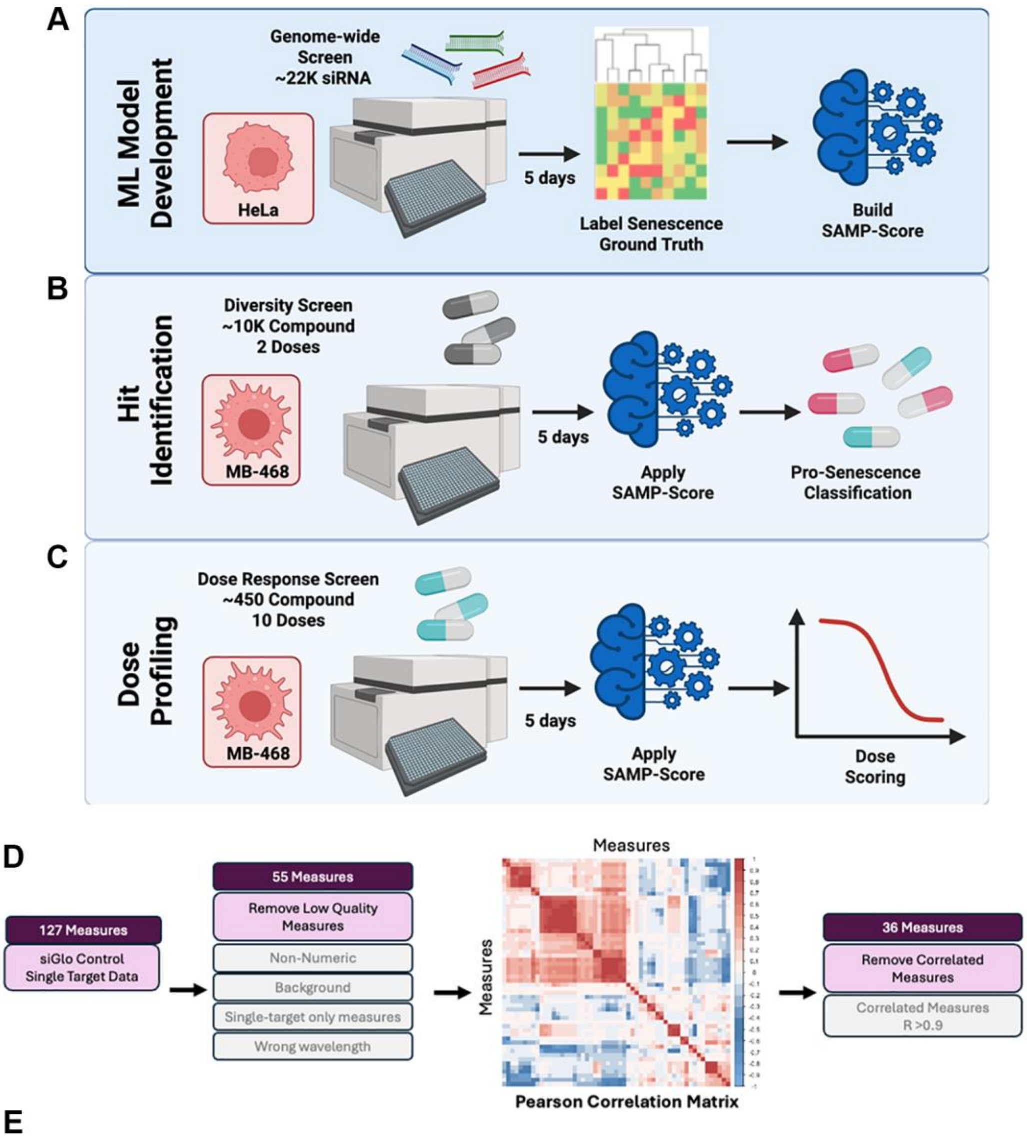

E

Measures Retained								Measures Removed			
1	Nuclei.Form.Factor	11	Nuclei.Neighbor.Count..SOI..	21	Cells.Area	31	Cells.Max.Intensity	1	Cells.Gyration.Radius	11	Nuclei.Entropy
2	Nuclei.Elongation	12	Nuclei.Spacing..MIN..	22	Cells.Form.Factor.	32	Cells.Major.Axis	2	Cells.Diameter	12	Cells.Entropy
3	Nuclei.Compactness	13	Nuclei.Neighbor.Count..MIN.	23	Cells.Elongation	33	Cells.Minor.Axis	3	Cells.Perimeter	13	Cells.Intensity.CV..Cell..
4	Nuclei.Chord.Ratio	14	Nuclei.Spacing..Gabriel..	24	Cells.Compactness	34	Cells.Skewness	4	Cells.Light.Flux.	14	Cells.Nuc.Cyto.Intensity.
5	Nuclei.Displacement	15	Nuclei.Neighbor.Count..Gabriel..	25	Cells.Chord.Ratio.	35	Cells.Kurtosis	5	Nuclei.Light.Flux.	15	Cells.Total.Intensity..Cell..
6	Nuclei.Intensity	16	Nuclei.Spacing..Lune..	26	Cells.Nuc.Cell.Area	36	Cells.Energy	6	Nuclei.Diameter	16	Cells.Intensity.SD..Cell.
7	Nuclei.Total.Intensity	17	Nuclei.Neighbor.Count..Lune..	27	Cells.Total.Intensity..Cyto..			7	Nuclei.Major.Axis	17	Cells.Intensity..Cyto.
8	Nuclei.Intensity.CV	18	Nuclei.Skewness	28	Cells.Intensity.CV..Cyto..			8	Nuclei.Gyration.Radius	18	Cells.Intensity..Cell.
9	Nuclei.Major.Axis.Angle	19	Nuclei.Kurtosis	29	Cells.Intensity.Spreading.			9	Nuclei.Perimeter	19	Nuclei.Intensity.SD
10	Nuclei.Spacing..SOI..	20	Nuclei.Energy	30	Cells.Intensity.SD..Cyto.			10	Nuclei.Area		

Supplementary Figure 1. High content screening overview and feature selection. (**A**–**C**) Overview of high three high content screens ((**A**) genome-wide siRNA screen in HeLa cells to establish ground truth for model development; (**B**) compound diversity screen for senescent hit identification; (**C**) dose profiling of senescent hits). (**D**) Feature dimensionality reduction strategy via Pearson correlation assessment. (**E**) List of final features which comprise SAMP profiles.

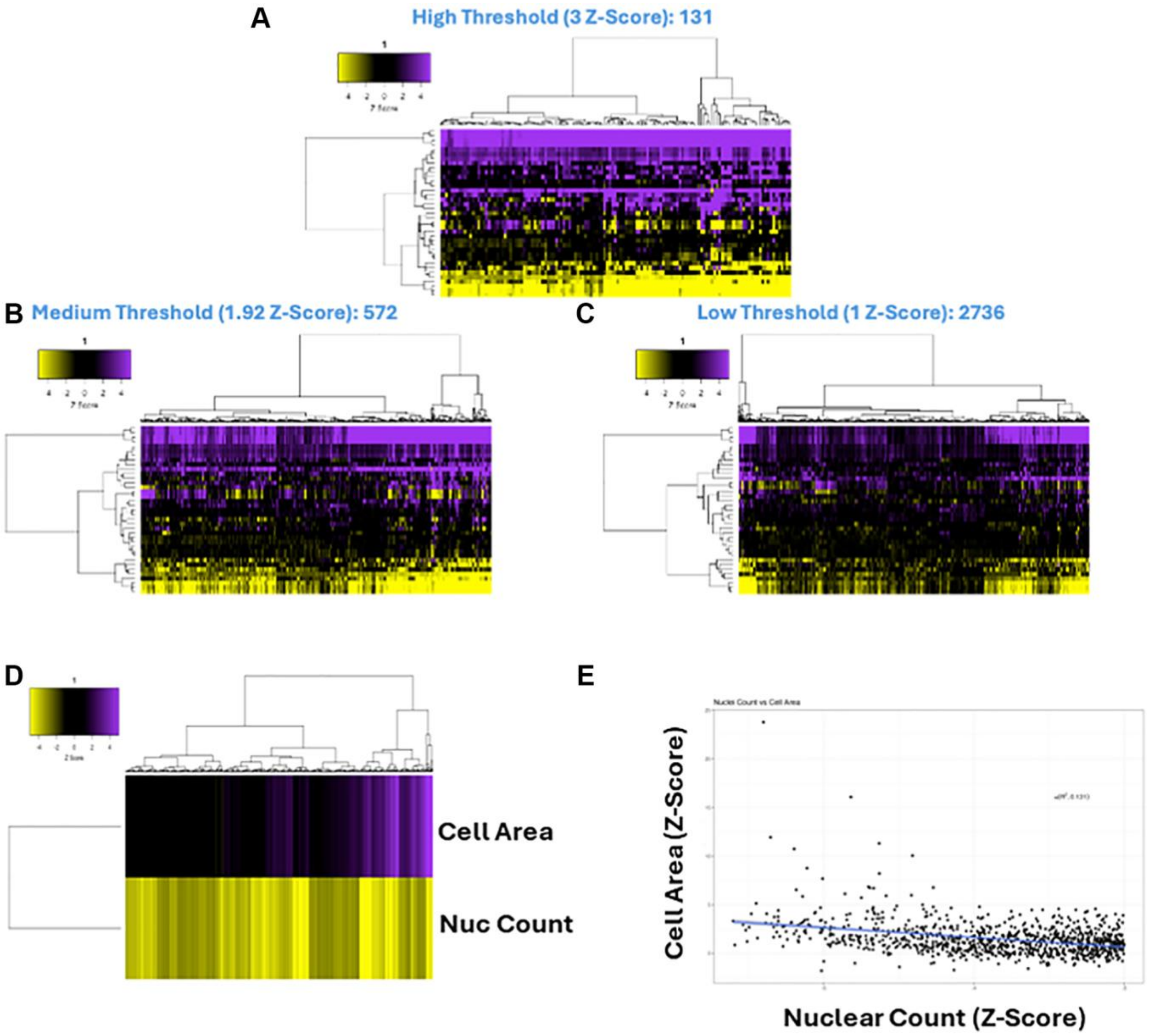

Supplementary Figure 2. Genome-wide siRNA HeLa screen – traditional screening readouts. (**A**–**C**) Heatmaps representing high content analysis feature (HCA; y-axis) profiles of a genome-wide siRNA screen treatments (siRNAs; x-axis) that reduce nuclear count and increase nuclear area by high, medium and low stringency thresholds. (**D**) Heatmap profile showing cell area and nuclear count Z-scores for all treatments. (**E**) Scatter plot showing nuclear count vs cell area Z-scores. In all heatmaps, purple indicates positive modulation and yellow negative modulation of greater than 1.96 Z-scores from siGLO control. Black indicates no change.

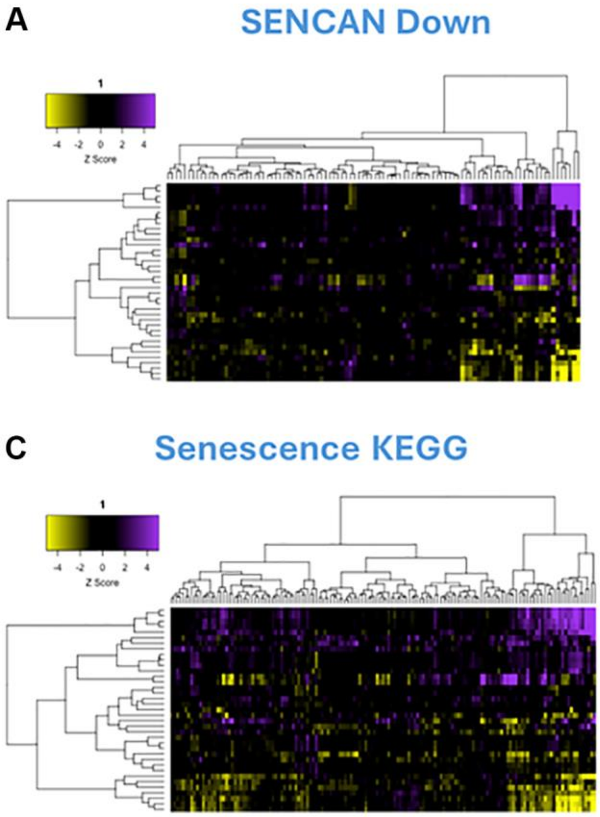

Supplementary Figure 3. Genome-wide siRNA HeLa screen – biased pathway labelling. (**A**–**D**) Heatmaps representing high content analysis feature (HCA; y-axis) profiles of a genome-wide siRNA screen treatments (siRNAs; x-axis) selected through identification as downregulated in SENCAN and SENESCopedia database or Senescence/Cell Cycle KEGG pathway analysis terms. In all heatmaps, purple indicates positive modulation and yellow negative modulation of greater than 1.96 Z-scores from DMSO vehicle control. Black indicates no change.

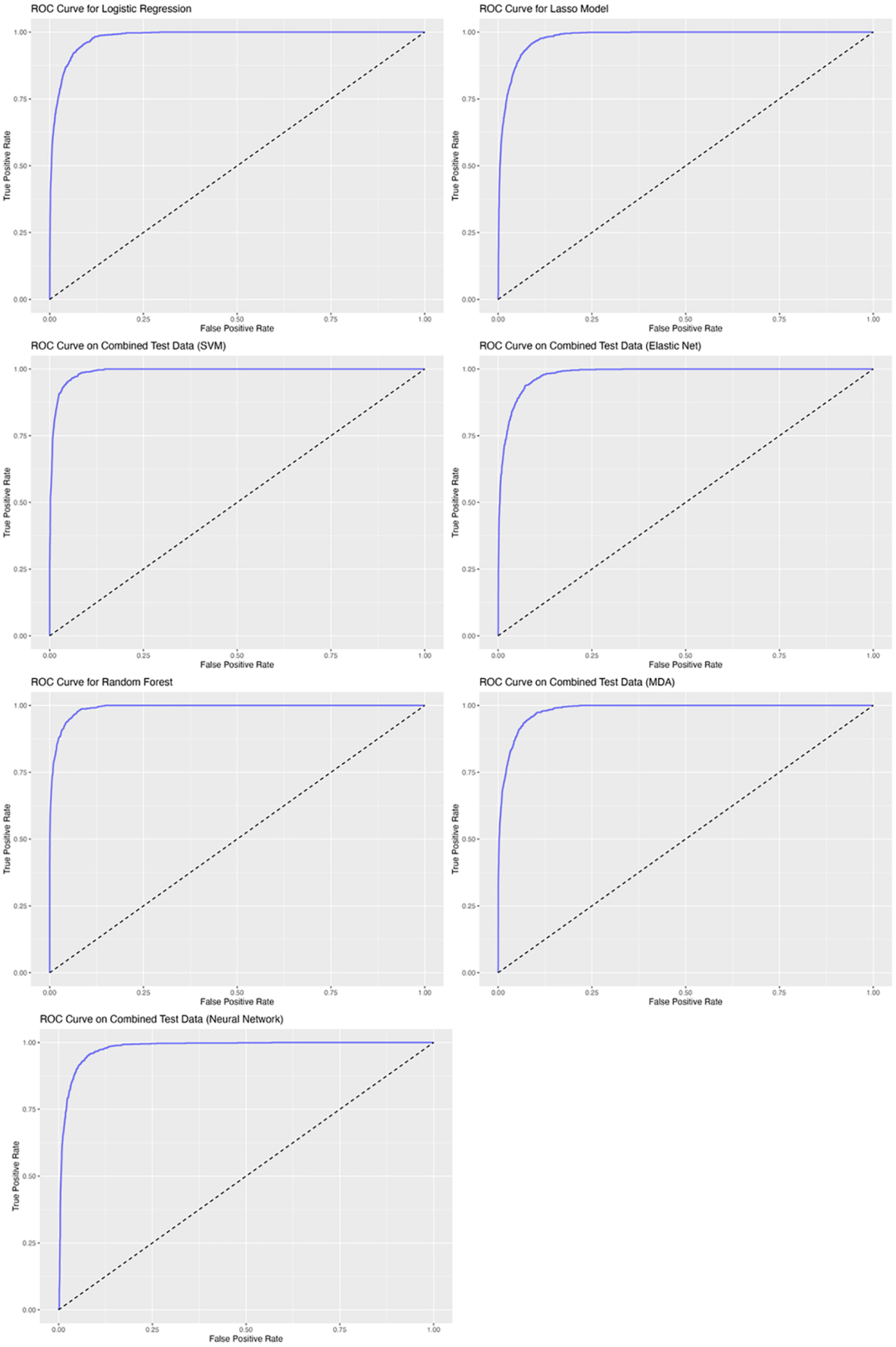

Supplementary Figure 4. Receiver operating characteristic (ROC) curves for all composite models of SAMP-Score.

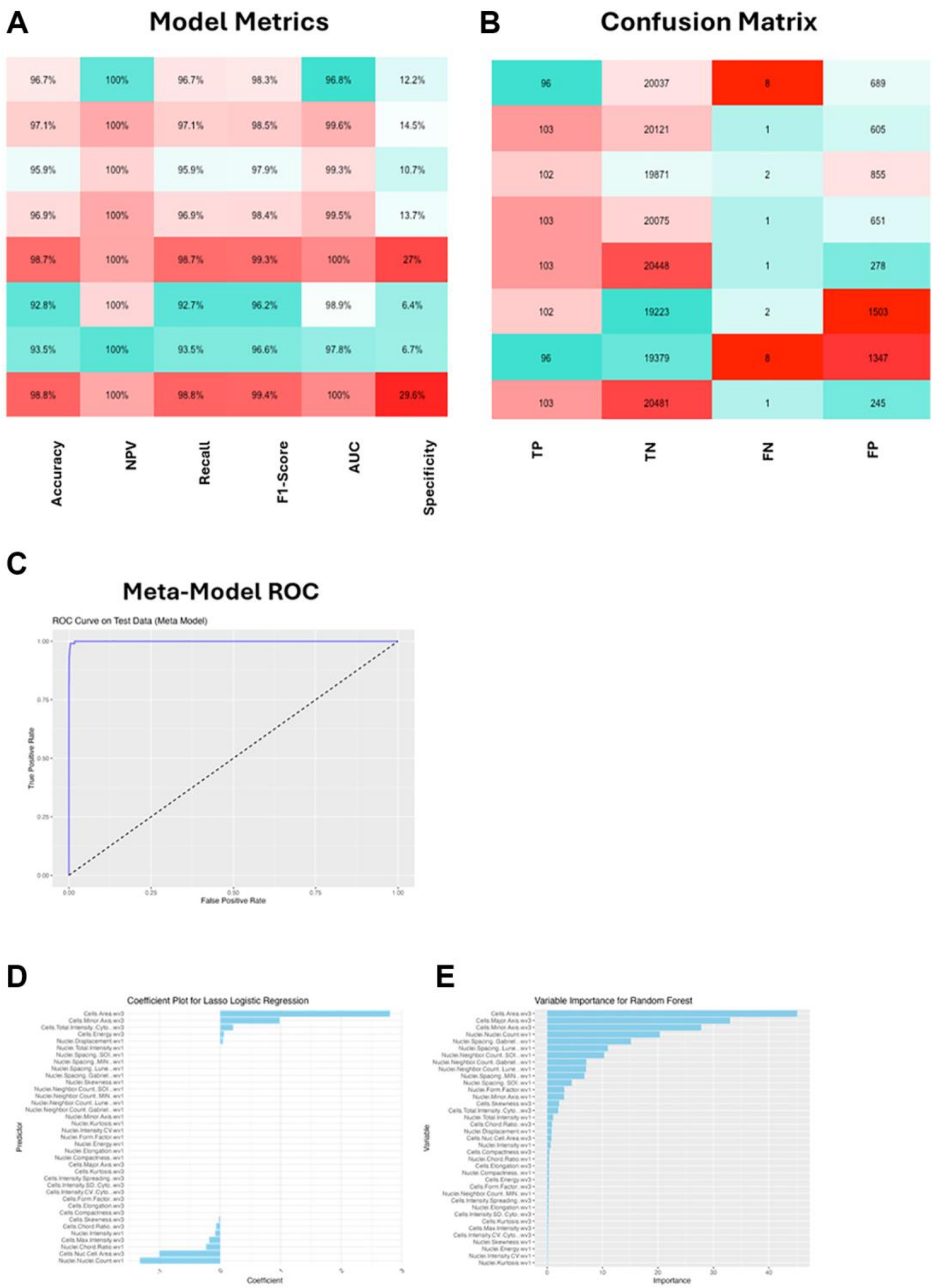

Supplementary Figure 5. Model Development and Metric Assessment using traditional threshold-based senescence labelling of training data. (**A**, **B**) Machine learning (ML) model assessment metrics and confusion matrix for all individual ML models and stacked meta-model (SAMP-Score). Abbreviations: NPV: Negative Prediction Value; AUC: Area under curve; PPV: Positive Prediction Value; TP: True Positive; TN: True Negative; FN: False Negative; FP: False Positive. (**C**) Receiver operating characteristic (ROC) curve for stacked meta model. (**D**, **E**) Model feature contributions to Lasso and Random Forest models.

A

		QM0005928
		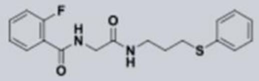
Toxicity	HepG2 pIC50	4.4
Solubility	ReaSol (uM)	180
DMPK	Clearance (ml/min/g)	8.3
	MDCK (Papp, nm/s)	235
Physiochemical Properties	MWt	346.42
	logP/CHI-logD	2.6/2.6
	tPSA	58
Metrics	LE/LLE	0.30/ 2.6
	IFI	0.1

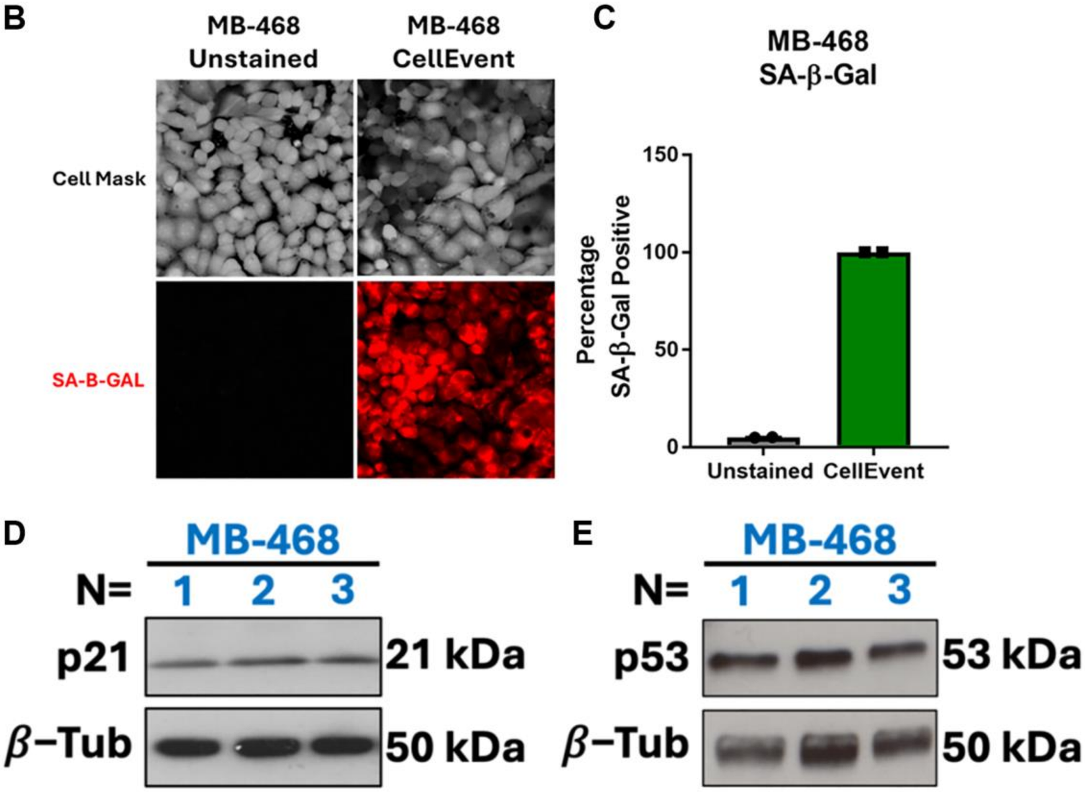

Supplementary Figure 6. QM0005928 physicochemical properties and SenMark+ cancer cell senescence markers. (**A**) Standard chemical assessment panel for QM5928. (**B**, **C**) Senescence-associated beta galactosidase staining of MB-468 cells. *N* = 1 with 2 technical replicates. (**D**, **E**) Immunoblotting of MB-468 cells for p21 and p53. *N* = 3.

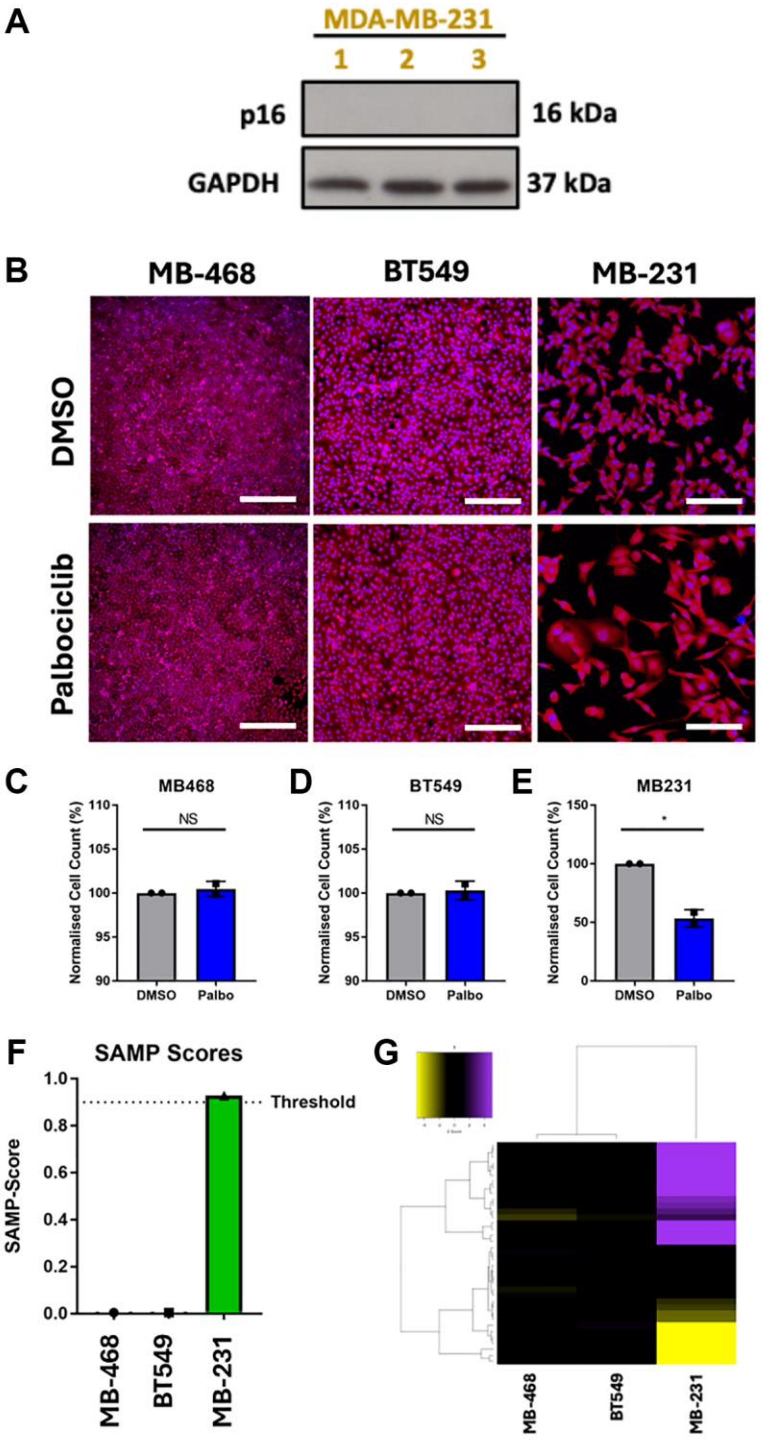

Supplementary Figure 7. SAMP-Score assessment of BLBC response to CDK Inhibition. (**A**) Immunoblotting of MB-231 cells for p16. N-3. (**B**–**E**) Response to 1 uM palbociclib treatment or vehicle control (DMSO) in MB-468, BT549 and MB-231 BLBC lines. (**F**) SAMP-Score classification coefficient for MB-468, BT549 and MB-231 BLBC lines treated with palbociclib. (**G**) Heatmap representing high content analysis feature (HCA; y-axis) profiles of MB-468, BT549 and MB-231 BLBC lines treated with palbociclib (x-axis). In all heatmaps, purple indicates positive modulation and yellow negative modulation of greater than 1.96 Z-scores from DMSO vehicle control. Black indicates no change.
