# Supplementary material for: SAMP-Score: a morphology-based machine learning classification method for screening pro-senescence compounds in p16 positive cancer cells
Source: Aging (Albany NY). 2025 Oct 30;17(11):2688–716. doi: 10.18632/aging.206333 (PMC12822720; doi:10.18632/aging.206333)
Supplement: Supplementary Data 1-9 [file aging-17-11-206333-s013.html]

9\_Testing\_Compound\_Screen\_2\_Clusters.knit


# 1. Define Ground Truth

```
library("gplots")
```

```
## 
## Attaching package: 'gplots'
```

```
## The following object is masked from 'package:stats':
## 
##     lowess
```

```
library("RColorBrewer")
library("matrixStats")
library("plyr")
```

```
## 
## Attaching package: 'plyr'
```

```
## The following object is masked from 'package:matrixStats':
## 
##     count
```

```
library("dplyr")
```

```
## 
## Attaching package: 'dplyr'
```

```
## The following objects are masked from 'package:plyr':
## 
##     arrange, count, desc, failwith, id, mutate, rename, summarise,
##     summarize
```

```
## The following object is masked from 'package:matrixStats':
## 
##     count
```

```
## The following objects are masked from 'package:stats':
## 
##     filter, lag
```

```
## The following objects are masked from 'package:base':
## 
##     intersect, setdiff, setequal, union
```

```
library("data.table")
```

```
## 
## Attaching package: 'data.table'
```

```
## The following objects are masked from 'package:dplyr':
## 
##     between, first, last
```

```
library("stringr")
library("ggplot2")
library(glmnet)
```

```
## Loading required package: Matrix
```

```
## Loaded glmnet 4.1-8
```

```
library(caret)
```

```
## Loading required package: lattice
```

```
library(pROC)
```

```
## Type 'citation("pROC")' for a citation.
```

```
## 
## Attaching package: 'pROC'
```

```
## The following objects are masked from 'package:stats':
## 
##     cov, smooth, var
```

```
library(e1071)  # Contains functions for SVM
library(randomForest)
```

```
## randomForest 4.7-1.1
```

```
## Type rfNews() to see new features/changes/bug fixes.
```

```
## 
## Attaching package: 'randomForest'
```

```
## The following object is masked from 'package:ggplot2':
## 
##     margin
```

```
## The following object is masked from 'package:dplyr':
## 
##     combine
```

```
library(mda)
```

```
## Loading required package: class
```

```
## Loaded mda 0.5-4
```

```
library(neuralnet)
```

```
## 
## Attaching package: 'neuralnet'
```

```
## The following object is masked from 'package:dplyr':
## 
##     compute
```

```
# Upload your file 
all<- read.csv("All_Data_Clusters_Consensus.csv")
all <- all[,-c(1)]
```

# 2. Data Partitioning

```
# Load required libraries
library(ROSE)  # For undersampling and oversampling
```

```
## Loaded ROSE 0.0-4
```

```
library(caret)  # For creating data partitions
library(dplyr)  # For data manipulation

#Tidy Variables
All_Tidy <- all[,-c(1:5)]

# Step 1: Split the data into train and test sets (80-20 split)
set.seed(123)  # For reproducibility
trainIndex <- createDataPartition(All_Tidy$Senescence_Status, p = 0.8, list = FALSE)
trainData <- All_Tidy[trainIndex,]
testData <- All_Tidy[-trainIndex,]

# Add a unique identifier to the training data
trainData <- trainData %>%
  ungroup() %>%
  mutate(id = row_number())

# Checking the distribution of the target variable in train and test sets
cat("Distribution in Training Set:\n")
```

```
## Distribution in Training Set:
```

```
print(table(trainData$Senescence_Status))
```

```
## 
## NonSen    Sen 
##  13336   3992
```

```
cat("Distribution in Test Set:\n")
```

```
## Distribution in Test Set:
```

```
print(table(testData$Senescence_Status))
```

```
## 
## NonSen    Sen 
##   3333    997
```

```
# Step 2: Perform undersampling to balance the classes
set.seed(123)
undersample_result <- ovun.sample(Senescence_Status ~ ., data = trainData, method = "under", p = 0.5)$data

# Checking the distribution after undersampling
cat("Distribution after Undersampling:\n")
```

```
## Distribution after Undersampling:
```

```
print(table(undersample_result$Senescence_Status))
```

```
## 
## NonSen    Sen 
##   3952   3992
```

```
# Step 3: Track and Extract Removed Observations
# Identify removed observations
# Removed IDs are those in trainData but not in undersample_result
removed_ids <- setdiff(trainData$id, undersample_result$id)

# Extract removed observations from the original training data
recycled_data <- trainData %>% filter(id %in% removed_ids)

# Remove the unique identifier column from all datasets
trainData <- trainData %>% select(-id)
undersample_result <- undersample_result %>% select(-id)
recycled_data <- recycled_data %>% select(-id)

# Combine recycled data with the test data
combined_testData <- bind_rows(testData, recycled_data)

# Checking the distribution in the combined test set
cat("Distribution in Combined Test Set:\n")
```

```
## Distribution in Combined Test Set:
```

```
print(table(combined_testData$Senescence_Status))
```

```
## 
## NonSen    Sen 
##  12717    997
```

```
# Split the Undersampled Training Data into Training and Validation
set.seed(123)
trainIndex_under <- createDataPartition(undersample_result$Senescence_Status, p = 0.5, list = FALSE)
trainData_part1 <- undersample_result[trainIndex_under,]
trainData_part2 <- undersample_result[-trainIndex_under,]

# Checking the distribution of the target variable in each part
cat("Distribution in Part 1 of Undersampled Data:\n")
```

```
## Distribution in Part 1 of Undersampled Data:
```

```
print(table(trainData_part1$Senescence_Status))
```

```
## 
## NonSen    Sen 
##   1976   1996
```

```
cat("Distribution in Part 2 of Undersampled Data:\n")
```

```
## Distribution in Part 2 of Undersampled Data:
```

```
print(table(trainData_part2$Senescence_Status))
```

```
## 
## NonSen    Sen 
##   1976   1996
```

```
#Replace Test Data With New Unseen Data
combined_testData <- read.csv("All_Compound_Scores.csv")
combined_testData <- combined_testData %>%
  filter(across(where(is.numeric), ~ !is.na(.) & is.finite(.)))
```

```
## Warning: Using `across()` in `filter()` was deprecated in dplyr 1.0.8.
## ℹ Please use `if_any()` or `if_all()` instead.
## Call `lifecycle::last_lifecycle_warnings()` to see where this warning was
## generated.
```

```
Indexed_Compounds <- combined_testData[,c(1:2)]
combined_testData <- combined_testData [,-c(1:2)]

column_names<- colnames(trainData_part1)

colnames(combined_testData) <- column_names[-38]
```

# 3. Lasso Regularisation

```
# Load required libraries
library(glmnet)
library(caret)
library(pROC)
library(ggplot2)

# Ensure Senescence_Status is a factor (for classification)
trainData_part1$Senescence_Status <- as.factor(trainData_part1$Senescence_Status)
trainData_part2$Senescence_Status <- as.factor(trainData_part2$Senescence_Status)

# Prepare training and test data for Lasso
x_train <- as.matrix(trainData_part1[, -which(names(trainData_part1) == "Senescence_Status")])
y_train <- trainData_part1$Senescence_Status
x_test <- as.matrix(combined_testData)

# Fit the Lasso model on trainData_part1
set.seed(123)
lasso_model <- glmnet(x_train, y_train, family = "binomial", alpha = 1)

# Use cross-validation to determine the best lambda
cv_model <- cv.glmnet(x_train, y_train, family = "binomial", alpha = 1)
```

```
## Warning: from glmnet C++ code (error code -48); Convergence for 48th lambda
## value not reached after maxit=100000 iterations; solutions for larger lambdas
## returned
```

```
## Warning: from glmnet C++ code (error code -50); Convergence for 50th lambda
## value not reached after maxit=100000 iterations; solutions for larger lambdas
## returned
```

```
best_lambda <- cv_model$lambda.min  # or cv_model$lambda.1se for simplicity

# Refit the model using the best lambda value
final_model <- glmnet(x_train, y_train, family = "binomial", alpha = 1, lambda = best_lambda)

# Make predictions on trainData_part2 using the Lasso model
predicted_probabilities_part2 <- predict(final_model, newx = as.matrix(trainData_part2[, -which(names(trainData_part2) == "Senescence_Status")]), s = best_lambda, type = "response")
predicted_classes_part2 <- ifelse(predicted_probabilities_part2 > 0.5, "Sen", "NonSen")

# Create the meta-model training data frame
lasso_meta_model_data_training <- data.frame(
  Lasso_Prob = as.vector(predicted_probabilities_part2),
  Senescence_Status = trainData_part2$Senescence_Status,
  Predicted_Status = predicted_classes_part2
)

# Save the meta-model training data for further use
write.csv(lasso_meta_model_data_training, "lasso_meta_model_data_training.csv", row.names = FALSE)

## Now we are going to apply the model to testing data and evaluate

# Make predictions on testing data using the Lasso model
predicted_probabilities_test <- predict(final_model, newx = x_test, s = best_lambda, type = "response")
predicted_classes_test <- ifelse(predicted_probabilities_test > 0.5, "Sen", "NonSen")

# Create the meta-model testing data frame
lasso_meta_model_data_testing <- data.frame(
  Lasso_Prob = as.vector(predicted_probabilities_test),
  Predicted_Status = predicted_classes_test
)

# Save the meta-model testing data for further use
write.csv(lasso_meta_model_data_testing, "lasso_meta_model_data_testing.csv", row.names = FALSE)
```

# 4. Logistic Regression

```
# Load required libraries
library(caret)
library(pROC)
library(ggplot2)
library(glmnet)

# Ensure Senescence_Status is a factor (for classification)
trainData_part1$Senescence_Status <- as.factor(trainData_part1$Senescence_Status)
trainData_part2$Senescence_Status <- as.factor(trainData_part2$Senescence_Status)

# Prepare training and test data for Logistic Regression
x_train <- as.matrix(trainData_part1[, -which(names(trainData_part1) == "Senescence_Status")])
y_train <- trainData_part1$Senescence_Status
x_test <- as.matrix(combined_testData)

# Fit the logistic regression model on trainData_part1
set.seed(123)
model_binary <- glm(Senescence_Status ~ ., data = trainData_part1, family = "binomial")
```

```
## Warning: glm.fit: fitted probabilities numerically 0 or 1 occurred
```

```
# Make predictions on trainData_part2 using the logistic regression model
predicted_probabilities_part2 <- predict(model_binary, newdata = trainData_part2, type = "response")
predicted_classes_part2 <- ifelse(predicted_probabilities_part2 > 0.5, "Sen", "NonSen")

# Create the meta-model training data frame
logistic_meta_model_data_training <- data.frame(
  logistic_Prob = as.vector(predicted_probabilities_part2),
  Senescence_Status = trainData_part2$Senescence_Status,
  Predicted_Status = predicted_classes_part2
)

# Save the meta-model training data for further use
write.csv(logistic_meta_model_data_training, "logistic_meta_model_data_training.csv", row.names = FALSE)

## Now we are going to apply the model to testing data and evaluate

# Make predictions on testing data using the logistic regression model
predicted_probabilities_test <- predict(model_binary, newdata = combined_testData, type = "response")
predicted_classes_test <- ifelse(predicted_probabilities_test > 0.5, "Sen", "NonSen")

# Create the meta-model testing data frame
logistic_meta_model_data_testing <- data.frame(
  logistic_Prob = as.vector(predicted_probabilities_test),
  Predicted_Status = predicted_classes_test
)

# Save the meta-model testing data for further use
write.csv(logistic_meta_model_data_testing, "logistic_meta_model_data_testing.csv", row.names = FALSE)
```

# 5. Elastic Nets

```
set.seed(123)

# Load required libraries
library(glmnet)
library(caret)
library(pROC)
library(ggplot2)

# Ensure Senescence_Status is a factor (for classification)
trainData_part1$Senescence_Status <- as.factor(trainData_part1$Senescence_Status)
trainData_part2$Senescence_Status <- as.factor(trainData_part2$Senescence_Status)

# Prepare training and test data for Elastic Net
x_train <- as.matrix(trainData_part1[, -which(names(trainData_part1) == "Senescence_Status")])
y_train <- trainData_part1$Senescence_Status

# Ensure the test data has the same columns as the training data
x_test <- as.matrix(combined_testData)

# Fit the Elastic Net model on trainData_part1
elastic_net_model <- cv.glmnet(x_train, y_train, family = "binomial", alpha = 0.5)

# Use cross-validation to determine the best lambda
best_lambda <- elastic_net_model$lambda.min  

# Refit the model using the best lambda value
final_elastic_net_model <- glmnet(x_train, y_train, family = "binomial", alpha = 0.5, lambda = best_lambda)

# Make predictions on trainData_part2 using the Elastic Net model
x_train_part2 <- as.matrix(trainData_part2[, colnames(x_train)])  # Ensure same columns
predicted_probabilities_part2 <- predict(final_elastic_net_model, newx = x_train_part2, s = best_lambda, type = "response")
predicted_classes_part2 <- ifelse(predicted_probabilities_part2 > 0.5, "Sen", "NonSen")

# Create the meta-model training data frame
en_meta_model_data_training <- data.frame(
  en_Probabilities = as.vector(predicted_probabilities_part2),
  Senescence_Status = trainData_part2$Senescence_Status,
  Predicted_Status = predicted_classes_part2
)

# Save the meta-model training data for further use
write.csv(en_meta_model_data_training, "en_meta_model_data_training.csv", row.names = FALSE)

## Now we are going to apply the model to testing data and evaluate

# Make predictions on testing data using the Elastic Net model
predicted_probabilities_test <- predict(final_elastic_net_model, newx = x_test, s = best_lambda, type = "response")
predicted_classes_test <- ifelse(predicted_probabilities_test > 0.5, "Sen", "NonSen")

# Create the meta-model testing data frame
en_meta_model_data_testing <- data.frame(
  en_Probabilities = as.vector(predicted_probabilities_test),
  Predicted_Status = predicted_classes_test
)

# Save the meta-model testing data for further use
write.csv(en_meta_model_data_testing, "en_meta_model_data_testing.csv", row.names = FALSE)
```

#6. Random Forrest

```
# Load required libraries
library(randomForest)
library(caret)
library(pROC)
library(ggplot2)

# Ensure Senescence_Status is a factor (for classification)
trainData_part1$Senescence_Status <- as.factor(trainData_part1$Senescence_Status)
trainData_part2$Senescence_Status <- as.factor(trainData_part2$Senescence_Status)

# Fit the Random Forest model on trainData_part1
set.seed(123)
rf_model <- randomForest(x = trainData_part1[, -which(names(trainData_part1) == "Senescence_Status")],
                         y = trainData_part1$Senescence_Status,
                         ntree = 500,
                         type = "classification")

# Make predictions on trainData_part2 using the Random Forest model
rf_predictions_part2 <- predict(rf_model, newdata = trainData_part2)

# Get class probabilities for the positive class
rf_probabilities_part2 <- predict(rf_model, newdata = trainData_part2, type = "prob")[, "Sen"]

# Create the meta-model training data frame
rf_meta_model_data_training <- data.frame(
  rf_Prob = as.vector(rf_probabilities_part2),
  Senescence_Status = trainData_part2$Senescence_Status,
  Predicted_Status = rf_predictions_part2
)

# Save the meta-model training data for further use
write.csv(rf_meta_model_data_training, "rf_meta_model_data_training.csv", row.names = FALSE)

## Now we are going to apply the model to testing data and evaluate 

# Make predictions on testing data using the Random Forest model
rf_predictions_test <- predict(rf_model, newdata = combined_testData)

# Get class probabilities for the positive class on testing data
rf_probabilities_test <- predict(rf_model, newdata = combined_testData, type = "prob")[, "Sen"]


# Create the meta-model testing data frame
rf_meta_model_data_testing <- data.frame(
  rf_Prob = as.vector(rf_probabilities_test),
  Predicted_Status = rf_predictions_test
)

# Save the meta-model testing data for further use
write.csv(rf_meta_model_data_testing, "rf_meta_model_data_testing.csv", row.names = FALSE)
```

# 7. SVMs

```
set.seed(123)

# Load required libraries
library(e1071)
library(caret)
library(pROC)
library(ggplot2)

# Ensure Senescence_Status is a factor (for classification)
trainData_part1$Senescence_Status <- as.factor(trainData_part1$Senescence_Status)
trainData_part2$Senescence_Status <- as.factor(trainData_part2$Senescence_Status)

# Prepare training and test data for SVM
x_train <- trainData_part1[, -which(names(trainData_part1) == "Senescence_Status")]
y_train <- trainData_part1$Senescence_Status
x_test <- combined_testData

# Fit the SVM model on trainData_part1
svm_model <- svm(x_train, y_train, kernel = "radial", probability = TRUE)

# Ensure trainData_part2 and combined_testData have the same features as x_train
x_train_part2 <- trainData_part2[, colnames(x_train), drop = FALSE]

# Make predictions on trainData_part2 using the SVM model
svm_predictions_part2 <- predict(svm_model, newdata = x_train_part2, probability = TRUE)
svm_probabilities_part2 <- attr(svm_predictions_part2, "probabilities")[, "Sen"]
svm_predicted_classes_part2 <- ifelse(svm_probabilities_part2 > 0.5, "Sen", "NonSen")

# Create the meta-model training data frame
svm_meta_model_data_training <- data.frame(
  svm_Prob = as.vector(svm_probabilities_part2),
  Senescence_Status = as.factor(trainData_part2$Senescence_Status),
  Predicted_Status = svm_predicted_classes_part2
)

# Save the meta-model training data for further use
write.csv(svm_meta_model_data_training, "svm_meta_model_data_training.csv", row.names = FALSE)

## Now we are going to apply the model to testing data and evaluate

# Make predictions on combined_testData using the SVM model
x_test_combined <- combined_testData
svm_predictions_test <- predict(svm_model, newdata = x_test_combined, probability = TRUE)
svm_probabilities_test <- attr(svm_predictions_test, "probabilities")[, "Sen"]
svm_predicted_classes_test <- ifelse(svm_probabilities_test > 0.5, "Sen", "NonSen")

# Create the meta-model testing data frame
svm_meta_model_data_testing <- data.frame(
  svm_Prob = as.vector(svm_probabilities_test),
  Predicted_Status = svm_predicted_classes_test
)

# Save the meta-model testing data for further use
write.csv(svm_meta_model_data_testing, "svm_meta_model_data_testing.csv", row.names = FALSE)
```

# 8. MDA

```
set.seed(123)

# Load required libraries
library(mda)
library(caret)
library(pROC)
library(ggplot2)

# Ensure Senescence_Status is a factor (for classification)
trainData_part1$Senescence_Status <- as.factor(trainData_part1$Senescence_Status)
trainData_part2$Senescence_Status <- as.factor(trainData_part2$Senescence_Status)

# Prepare training data for MDA
x_train_part2 <- trainData_part2[, -which(names(trainData_part2) == "Senescence_Status")]
y_train_part2 <- trainData_part2$Senescence_Status

# Fit the MDA model on trainData_part2
mda_model <- mda(Senescence_Status ~ ., data = trainData_part2)

# Ensure test data has the same features as x_train_part2
x_test_combined <- combined_testData

# Make predictions on trainData_part2 using the MDA model
predicted_probabilities_train_mda <- predict(mda_model, newdata = trainData_part2, type = "posterior")
predicted_classes_train_mda <- ifelse(predicted_probabilities_train_mda[, "Sen"] > 0.5, "Sen", "NonSen")

# Create and save the meta-model data frame for trainData_part2
mda_meta_model_training <- data.frame(
  mda_Prob = as.vector(predicted_probabilities_train_mda[, "Sen"]),
  Senescence_Status = as.factor(trainData_part2$Senescence_Status),
  Predicted_Status = predicted_classes_train_mda
)
write.csv(mda_meta_model_training, "mda_meta_model_training.csv", row.names = FALSE)

## Now we are going to apply the model to combined_testData and evaluate

# Make predictions on combined_testData using the MDA model
predicted_probabilities_test_mda <- predict(mda_model, newdata = combined_testData, type = "posterior")
predicted_classes_test_mda <- ifelse(predicted_probabilities_test_mda[, "Sen"] > 0.5, "Sen", "NonSen")

# Create and save the meta-model data frame for combined_testData
mda_meta_model_testing <- data.frame(
  mda_Prob = as.vector(predicted_probabilities_test_mda[, "Sen"]),
  Predicted_Status = predicted_classes_test_mda
)
write.csv(mda_meta_model_testing, "mda_meta_model_testing.csv", row.names = FALSE)
```

# 9. Neural Network

```
set.seed(123)

# Load required libraries
library(neuralnet)
library(caret)
library(pROC)
library(ggplot2)

# Prepare the data
Test_Train <- trainData_part1
Test_Test <- trainData_part2
Combined_Test <- combined_testData

# Convert Senescence_Status variable to binary {0, 1}
Test_Train$Senescence_Status <- ifelse(Test_Train$Senescence_Status == "Sen", 1, 0)
Test_Test$Senescence_Status <- ifelse(Test_Test$Senescence_Status == "Sen", 1, 0)

# Define formula for the neural network
formula <- as.formula("Senescence_Status ~ .")

# Train the neural network using trainData_part1
nn_model <- neuralnet(
  formula,
  data = Test_Train,
  hidden = c(20,10,5,2),  # Define the number of neurons in hidden layers
  linear.output = FALSE  # Use sigmoid activation function for binary classification
)

# Prepare meta-model training data
x_train_part2 <- Test_Test[, colnames(Test_Train), drop = FALSE]

# Make predictions on Test_Test using the NN model
predicted_probabilities_part2 <- compute(nn_model, as.matrix(x_train_part2[, -which(names(x_train_part2) == "Senescence_Status")]))$net.result
predicted_classes_part2 <- ifelse(predicted_probabilities_part2 > 0.5, 1, 0)

# Create and save the meta-model data frame for Test_Test
nn_meta_model_data_train <- data.frame(
  nn_Prob = as.vector(predicted_probabilities_part2),
  Senescence_Status = as.factor(Test_Test$Senescence_Status),
  Predicted_Status = factor(predicted_classes_part2, levels = c(0, 1), labels = c("NonSen", "Sen"))
)
write.csv(nn_meta_model_data_train, "nn_meta_model_training.csv", row.names = FALSE)

## Now we are going to apply the model to Combined_Test and evaluate

# Make predictions on Combined_Test using the NN model
x_test_combined <- Combined_Test
predicted_probabilities_test <- compute(nn_model, as.matrix(x_test_combined))$net.result
predicted_classes_test <- ifelse(predicted_probabilities_test > 0.5, 1, 0)

# Create and save the meta-model data frame for Combined_Test
nn_meta_model_data_test <- data.frame(
  nn_Prob = as.vector(predicted_probabilities_test),
  Predicted_Status = factor(predicted_classes_test, levels = c(0, 1), labels = c("NonSen", "Sen"))
)
write.csv(nn_meta_model_data_test, "nn_meta_model_testing.csv", row.names = FALSE)
```

# 10. Meta Model

```
set.seed(123)

# Load required libraries
library(glmnet)
library(caret)
library(pROC)
library(ggplot2)

# Combine model predictions and response variable for training
meta_model_data_train <- as.data.frame(cbind(
  lasso_meta_model_data_training[,1],
  svm_meta_model_data_training[,1],
  en_meta_model_data_training[,1],
  rf_meta_model_data_training[,1],
  mda_meta_model_training[,1],
  logistic_meta_model_data_training[,1],
  nn_meta_model_data_train[,1:2]
))

colnames(meta_model_data_train) <- c("lasso", "svm", "en", "rf", "mda", "logistic", "nn", "Senescence_Status")

# Extract the predictors and response for training
train_predictors <- meta_model_data_train[, -ncol(meta_model_data_train)]
train_response <- as.factor(meta_model_data_train$Senescence_Status)

# Fit the Lasso model on the training meta-model data
lasso_model <- cv.glmnet(
  x = as.matrix(train_predictors), 
  y = as.numeric(train_response) - 1,  # glmnet requires response to be zero-indexed
  family = "binomial", 
  alpha = 1
)

# Get the best lambda
best_lambda <- lasso_model$lambda.min

# Combine model predictions and response variable for testing
meta_model_data_test <- as.data.frame(cbind(
  lasso_meta_model_data_testing[,1],
  svm_meta_model_data_testing[,1],
  en_meta_model_data_testing[,1],
  rf_meta_model_data_testing[,1],
  mda_meta_model_testing[,1],
  logistic_meta_model_data_testing[,1],
  nn_meta_model_data_test[,1:2]
))

colnames(meta_model_data_test) <- c("lasso", "svm", "en", "rf", "mda", "logistic", "nn", "Senescence_Status")

# Extract the predictors and response for testing
test_predictors <- meta_model_data_test[, -ncol(meta_model_data_test)]
test_response <- as.factor(meta_model_data_test$Senescence_Status)

# Make predictions on the meta-model test data using the trained Lasso model
predicted_probabilities_test <- predict(lasso_model, newx = as.matrix(test_predictors), s = best_lambda, type = "response")
predicted_classes_test <- ifelse(predicted_probabilities_test > 0.9, "Sen", "NonSen")

predicted_classes_DDU_Screen <- cbind(Indexed_Compounds,predicted_classes_test,combined_testData)


write.csv(predicted_classes_DDU_Screen, "DDU_Screen_1_Predictions.csv")

# Logarithmic scatter plot
Log_Scatter_Plot <- ggplot(predicted_classes_DDU_Screen, aes(x = Nuclei.Nuclei.Count.wv1, y = Cells.Area.wv3, color = s1)) +
  geom_point(size = 0.1) +
  scale_y_log10() +
  labs(x = "Nuclei Count (Z Score)", y = "Cells Area (Log10 Z Score)", color = "Senescence Prediction") +
  geom_vline(xintercept = -1.92, linetype = "dotted", color = "blue", size = 1) +  # Vertical line at x = 1.92
  geom_hline(yintercept = 1.92, linetype = "dotted", color = "black", size = 1) +   # Horizontal line at y = 1.92
  theme_bw() +
  theme(text = element_text(size = 14))
```

```
## Warning: Using `size` aesthetic for lines was deprecated in ggplot2 3.4.0.
## ℹ Please use `linewidth` instead.
## This warning is displayed once every 8 hours.
## Call `lifecycle::last_lifecycle_warnings()` to see where this warning was
## generated.
```

```
# Save the plot
ggsave("Log_Scatter_Plot.png", plot = Log_Scatter_Plot, width = 8, height = 6)
```

```
## Warning in self$trans$transform(x): NaNs produced
```

```
## Warning: Transformation introduced infinite values in continuous y-axis
```

```
## Warning: Removed 1490 rows containing missing values (`geom_point()`).
```

```
# Non-logarithmic scatter plot
Scatter_Plot <- ggplot(predicted_classes_DDU_Screen, aes(x = Nuclei.Nuclei.Count.wv1, y = Cells.Area.wv3, color = s1)) +
  geom_point(size = 0.1) +
  labs(x = "Nuclei Count (Z Score)", y = "Cells Area (Z Score)", color = "Senescence Prediction") +
  theme_bw() +
  theme(text = element_text(size = 14))

# Save the plot
ggsave("Scatter_Plot.png", plot = Scatter_Plot, width = 8, height = 6)
```

# 11. Plot SAMP Scores

```
# Create a folder to store the outputs if it doesn't already exist
output_dir <- "SAMP_Score_Plots"
dir.create(output_dir, showWarnings = FALSE)

library(tidyr)
```

```
## 
## Attaching package: 'tidyr'
```

```
## The following objects are masked from 'package:Matrix':
## 
##     expand, pack, unpack
```

```
library(gplots)
library(ggplot2)

Heatmap_Data <- cbind(Indexed_Compounds,predicted_probabilities_test, combined_testData)

Heatmap <- Heatmap_Data[,c(1,2,3)]

Heatmap_adjusted <- spread(Heatmap, key = Concentration, value = s1)
Heatmap_adjusted <- Heatmap_adjusted %>%
  mutate(across(everything(), ~ifelse(is.na(.), 0, .)))

rownames(Heatmap_adjusted) <- Heatmap_adjusted[,1]
Heatmap_adjusted <- Heatmap_adjusted[,-1]

all_matrix <- as.matrix(Heatmap_adjusted)

# Create heatmap
breaks <- unique(c(seq(0,0.87,length=100),seq(0.87,0.89,length=100), seq(0.9,1,length=100)))
my_palette <- colorRampPalette(c("black","black","#D3EC01","#46D100"))(length(breaks)-1)

# Save heatmap to file
heatmap_filename <- file.path(output_dir, "Heatmap.png")
png(heatmap_filename, width = 800, height = 800)

heatmap.2(all_matrix,
          Rowv = TRUE,
          Colv = FALSE,
          col = my_palette,
          breaks = breaks,
          density.info = "none",
          trace = "none",
          dendrogram = c("row"), 
          symm = FALSE, symkey = FALSE, symbreaks = TRUE,
          labRow = FALSE,
          labCol = colnames(all_matrix),
          cexRow = 0.1,
          cexCol = 2,
          margins = c(8,2),
          key.title = "1", 
          key.xlab = "Z Score", 
          sepcolor = c("black"), 
          sepwidth = c(0.05, 0.05),
          distfun = function(x) dist(x, method = "euclidean"),
          hclust = function(x) hclust(x, method = "ward.D2"))

dev.off() # Close the PNG device
```

```
## quartz_off_screen 
##                 2
```

```
# Create scatter plot and save it to file
plot <- ggplot(Heatmap, aes(x = Concentration, y = s1)) +
  geom_point() +
  labs(x = "Variables", y = "SAMP-Score", title = "Scatter Plot of All Doses / Compounds") +
  theme(axis.text.x = element_text(angle = 45, hjust = 1))

plot_filename <- file.path(output_dir, "Scatter_Plot.png")
ggsave(plot_filename, plot = plot, width = 8, height = 6)
```

#12. Morphology Heatmaps

```
Heatmap_Data <- cbind(Indexed_Compounds,predicted_probabilities_test, combined_testData)

library(tidyr)
library(gplots) # For heatmap.2 function

# Create a folder to store the heatmaps if it doesn't already exist
dir.create("Morphology_Heatmaps", showWarnings = FALSE)

# Remove the s1 column (column 3) but keep columns 1 and 2
Heatmap <- Heatmap_Data[,-3]

# List of unique concentrations
concentrations <- unique(Heatmap$Concentration)

# Loop through each concentration to create and save a heatmap
for (conc in concentrations) {
  
  # Filter the data for the current concentration
  Heatmap_adjusted <- Heatmap[Heatmap$Concentration == conc, ]
  
  # Set row names to DDDNumber and remove the Concentration column
  rownames(Heatmap_adjusted) <- Heatmap_adjusted$DDDNumber
  Heatmap_adjusted <- Heatmap_adjusted[,-1]  # Remove Concentration column, keep DDDNumber as row names
  
  # Convert to matrix
  all_matrix <- as.matrix(Heatmap_adjusted[,-1])  # Exclude DDDNumber from the matrix
  
  # Define color palette and breaks
  breaks <- unique(c(seq(-5,-1,length=100),seq(-1,0.1,length=100), seq(1,5,length=100)))
  my_palette <- colorRampPalette(c("yellow","black","black","purple"))(length(breaks)-1)
  
  # Save the heatmap as a PNG file
  png_filename <- paste0("Morphology_Heatmaps/Heatmap_Concentration_", conc, ".png")
  png(png_filename, width = 800, height = 800)
  
  # Create heat map
  heatmap.2(t(all_matrix),
            Rowv = T,
            Colv = T,
            col=my_palette,
            breaks=breaks,
            density.info="none",
            trace="none",
            #main = "199 compounds; Length or Prev",
            dendrogram=c("both"), 
            symm=F,symkey=F,symbreaks=T,
            labRow=F,
            labCol=F,
            cexRow=0.8,
            cexCol=0.1,
            margins = c(8,2),
            key.title = "1" , key.xlab="Z Score", 
            #rowsep =c(0,4,12,19,31,37,43),
            #colsep =c(0,233),
            sepcolor = c("black"),sepwidth = c(0.05, 0.05),
            #ColSideColors=condition_colors, scale="none",
            distfun = function(x) dist(x, method = "euclidean"),
            hclust=function(x) hclust(x,method="ward.D2"))
  
  dev.off()  # Close the PNG device
}
```

#13 Morphology of QM0005928

```
Heatmap_Data <- cbind(Indexed_Compounds, predicted_probabilities_test, combined_testData)

library(tidyr)
library(gplots) # For heatmap.2 function

# Create a folder to store the heatmaps if it doesn't already exist
dir.create("Morphology_Heatmaps", showWarnings = FALSE)

# Remove the s1 column (column 3) but keep columns 1 and 2
Heatmap <- Heatmap_Data[,-3]

# Specify the compound you're interested in
compound_of_interest <- "QM0005928"  # Replace with the actual compound name

# Filter the data for the specified compound
Heatmap_adjusted <- Heatmap[Heatmap$DDDNumber == compound_of_interest, ]

# Check if the filtering resulted in data
if (nrow(Heatmap_adjusted) == 0) {
  stop("No data found for the specified compound.")
}

# Update the Concentration values and ensure it is numeric
Heatmap_adjusted$Concentration <- as.numeric(Heatmap_adjusted$Concentration) * 1000000

# Sort by Concentration
Heatmap_adjusted <- Heatmap_adjusted[order(Heatmap_adjusted$Concentration), ]

# Set row names to Concentration and remove the Concentration column
rownames(Heatmap_adjusted) <- Heatmap_adjusted$Concentration
Heatmap_adjusted <- Heatmap_adjusted[,-1]  # Remove Concentration column, keep it as row names

# Convert to matrix
all_matrix <- as.matrix(Heatmap_adjusted)

# Define color palette and breaks
breaks <- unique(c(seq(-5, -1, length=100), seq(-1, 0.1, length=100), seq(1, 5, length=100)))
my_palette <- colorRampPalette(c("yellow", "black", "black", "purple"))(length(breaks) - 1)

# Save the heatmap as a PNG file
png_filename <- paste0("Morphology_Heatmaps/Heatmap_Compound_", compound_of_interest, ".png")
png(png_filename, width = 800, height = 800)

# Create heat map
heatmap.2(t(all_matrix),
          Rowv = TRUE,
          Colv = NA,  # Turn off column clustering
          col = my_palette,
          breaks = breaks,
          density.info = "none",
          trace = "none",
          dendrogram = c("row"),  # Only cluster rows
          symm = FALSE, symkey = FALSE, symbreaks = TRUE,
          labRow = FALSE,
          labCol = rownames(all_matrix),
          cexRow = 0.8,
          cexCol = 2,
          margins = c(8, 2),
          key.title = "1", key.xlab = "Z Score",
          sepcolor = c("black"), sepwidth = c(0.05, 0.05),
          distfun = function(x) dist(x, method = "euclidean"),
          hclust = function(x) hclust(x, method = "ward.D2"))

dev.off()  # Close the PNG device
```

```
## quartz_off_screen 
##                 2
```

# Line Chart QM0005928

```
# Load necessary libraries
library(ggplot2)
library(dplyr)

# Create a folder to store the line graphs if it doesn't already exist
dir.create("Morphology_LineGraphs", showWarnings = FALSE)

# Remove the s1 column (column 3) but keep columns 1 and 2
Line_Chart_Data <- Heatmap_Data

# Specify the compound you're interested in
compound_of_interest <- "QM0005928"  # Replace with the actual compound name

# Filter the data for the specified compound
Line_Chart_Data_Adjusted<- Line_Chart_Data[Line_Chart_Data$DDDNumber == compound_of_interest, ]

# Check if the filtering resulted in data
if (nrow(Line_Chart_Data_Adjusted) == 0) {
  stop("No data found for the specified compound.")
}

# Update the Concentration values and ensure it is numeric
Line_Chart_Data_Adjusted$Concentration <- as.numeric(Line_Chart_Data_Adjusted$Concentration) * 1000000

# Sort by Concentration
Line_Chart_Data_Adjusted<- Line_Chart_Data_Adjusted[order(Line_Chart_Data_Adjusted$Concentration), ]

# Extract dose and s1 columns for plotting
dose_vs_s1 <- Line_Chart_Data_Adjusted %>%
  select(Concentration, s1)  # Ensure s1 is included

# Create a line plot of Dose vs s1
png_filename <- paste0("Morphology_LineGraphs/LineGraph_Compound_", compound_of_interest, ".png")
png(png_filename, width = 800, height = 600)

ggplot(dose_vs_s1, aes(x = log10(Concentration), y = s1)) +
  geom_line() +
  geom_point() +
  labs(title = paste("Dose vs SAMP-Score for Compound", compound_of_interest),
       x = "Log10 Dose (Scaled Concentration)",
       y = "SAMP-Score") +
  theme_bw() +  # Use black and white theme
  theme(
    text = element_text(size = 16),  # Increase text size for titles and labels
    axis.title = element_text(size = 14),  # Increase text size for axis titles
    axis.text = element_text(size = 12)   # Increase text size for axis labels
  )

dev.off()  # Close the PNG device
```

```
## quartz_off_screen 
##                 2
```
